# Functional Characterisation of Bile Metagenome: Study of Metagenomic Dark Matter

**DOI:** 10.3390/microorganisms9112201

**Published:** 2021-10-21

**Authors:** Carlos Sabater, Natalia Molinero, Manuel Ferrer, Carmen María García Bernardo, Susana Delgado, Abelardo Margolles

**Affiliations:** 1 Department of Microbiology and Biochemistry of Dairy Products, Instituto de Productos Lácteos de Asturias (IPLA), Consejo Superior de Investigaciones Científicas (CSIC), Paseo Río Linares S/N, 33300 Villaviciosa, Spain; natalia.molinero@csic.es (N.M.); sdelgado@ipla.csic.es (S.D.); amargolles@ipla.csic.es (A.M.); 2 Instituto de Investigación Sanitaria del Principado de Asturias (ISPA), 33011 Oviedo, Spain; 3 Instituto de Investigación en Ciencias de la Alimentación CIAL, (CSIC-UAM) CEI (UAM + CSIC), Nicolás Cabrera, 9, 28049 Madrid, Spain; 4 Institute of Catalysis, Marie Curie 2, 28049 Madrid, Spain; mferrer@icp.csic.es; 5 General Surgery Service, Central University Hospital of Asturias, 33011 Oviedo, Spain; cmgbernardo2@hotmail.com

**Keywords:** bile microbiome, metagenomic dark matter, multidrug resistance, molecular docking, molecular dynamics

## Abstract

Gallbladder metagenome involves a wide range of unidentified sequences comprising the so-called metagenomic dark matter. Therefore, this study aimed to characterise three gallbladder metagenomes and a fosmid library with an emphasis on metagenomic dark matter fraction. For this purpose, a novel data analysis strategy based on the combination of remote homology and molecular modelling has been proposed. According to the results obtained, several protein functional domains were annotated in the metagenomic dark matter fraction including acetyltransferases, outer membrane transporter proteins, membrane assembly factors, DNA repair and recombination proteins and response regulator phosphatases. In addition, one deacetylase involved in mycothiol biosynthesis was found in the metagenomic dark matter fraction of the fosmid library. This enzyme may exert a protective effect in Actinobacteria against bile components exposure, in agreement with the presence of multiple antibiotic and multidrug resistance genes. Potential mechanisms of action of this novel deacetylase were elucidated by molecular simulations, highlighting the role of histidine and aspartic acid residues. Computational pipelines presented in this work may be of special interest to discover novel microbial enzymes which had not been previously characterised.

## 1. Introduction

The human gallbladder comprises a scarcely studied microbial ecological niche compared to the human faecal microbiome. Current data are mainly limited to gallbladder samples resected during surgery [1]. Moreover, low bacterial biomass found in human bile samples makes the analysis complex. Up to date, few studies characterised the human biliary metagenome. Song et al. [2], performed metagenomic sequencing of the biliary microbiota of individuals who had undergone laparoscopic cholecystectomy or radical cholecystectomy. Shen et al. [3], reported metagenomic characterisation of bile samples from patients with gallstone disease. Previous works in our group were performed to characterise, by 16S rDNA and shotgun metagenomic sequencing, bile samples from the gallbladder of individuals suffering from lithiasis as well as subjects without any record of the hepatobiliary disorder [1]. We observed that bile metagenomes contain a larger number of unclassified sequences and protein functional domains than faecal samples from healthy individuals [1], comprising the so-called metagenomic dark matter. In this sense, the unravelling of taxonomically and functionally unassigned sequences in environmental and host metagenomes has been identified as a central priority and several studies to elucidate metagenomic dark matter in different microbial ecosystems have been performed [4,5,6,7]. However, there is a scarcity of bioinformatic pipelines to properly identify these unassigned sequences. Network analysis has been proposed to characterise and prioritise microbial dark matter in a variety of microbial ecosystems from all environments [8], while remote homology techniques and profile-profile comparisons of conservation patterns between sequence families have been used to elucidate unknown biological functions [9,10]. In addition, molecular modelling techniques have been employed for the structural solution of bacterial enzymes to uncover their true biochemical functions and enhanced the discovery of novel potential enzymes [11]. Recently, the utility of molecular simulations to integrate glycosidase sequences found in the genome of beneficial bacteria and their chemical interaction with specific oligosaccharide structures by reconstructing carbohydrate fermentation that takes place in the colon has also been reported [12]. These advanced computational chemistry methods could be of great interest for novel enzyme discovery although their applications to the microbial dark matter field have not been extensively developed yet.

Therefore, this study aimed to provide an exhaustive characterisation of bile metagenome with a strong emphasis on multiple drug resistance (MDR) and antibiotic resistance genes (ARGs), as well as those protein functional domains whose function remains unidentified, comprising the metagenomic dark matter fraction. Potential mechanisms of action of one of the most biologically relevant metagenomic dark matter domains have been elucidated through molecular modelling techniques.

## 2. Materials and Methods

### 2.1. Bile Samples and Participants

Human bile samples were obtained aseptically and directly from the gallbladder by surgeons at the General Surgery Service of HUCA (Central University Hospital of Asturias, Oviedo, Spain), during liver transplant surgery from liver donors without hepatobiliary disease, belonging to a previous study [1]. From the total of the participants of this study, 6 donors, who presented a high ratio of 16S/18S after total DNA extraction and quantification, were selected. Subjects were 3 male and 3 female and aged range among 49 to 68 years old, who had stated no more than 48 h stay in the intensive care unit (ICU) of the hospital’s before the transplant, and had not received antibiotics at ICU for more than 24 h.

The Bioethics Committee of CSIC (Consejo Superior de Investigaciones Científicas) and the Regional Ethics Committee for Clinical Research (Servicio de Salud del Principado de Asturias n°112/13) issued ethical approval for this study, in compliance with the Declaration of Helsinki of 1964. All experiments were carried out in accordance with the approved guidelines and regulations.

Bile samples from the 6 donors were split so that samples from 3 donors (codes H-04, H-05 and H-06 respectively) were used for functional analysis by shotgun metagenomics as previously reported [1]. DNA extraction yields from H-04, H-05 and H-06 samples ranged from 15 to 40 ng mL^−1^. On the other hand, samples from the remaining 3 donors (H-07, H-12 and H-13) were used to generate a fosmid library and the subsequent functional metagenomic study.

### 2.2. Fosmid Libraries Preparation

#### 2.2.1. Extraction of Total DNA for Library Construction

A pool of bile samples (16 mL in total) from 3 donors were used to prepare the fosmid libraries. In this regard, sample pooling reflects microbial diversity of the gallbladder and provides a general overview of metabolic capabilities of bile microbiota regardless of individual variability. To extract the total DNA, an optimised protocol previously described was used [1], with some modifications to obtain low fragmented DNA. Bile samples were centrifuged at maximum speed at room temperature for 10 min and pellets re-suspended in 0.5 mL of extraction buffer [200 mM Tris-HCl pH 7.0, 25 mM EDTA, 250 mM NaCl, 20 mg mL^−1^ lysozyme (Merck, Darmstadt, Germany), 5 μg mL^−1^ of lysostaphin (Sigma-Aldrich, Saint Louis, MO, USA), and 40 U mL^−1^ mutanolysin (Sigma-Aldrich, Saint Louis, MO, USA)]. Enzymatic lysis was performed for 1 h at 37 °C; after that, SDS was added to a final concentration of 0.5% (*w/v*), and the lysate solution was treated with 5 μL of RNase Cocktail^TM^ Enzyme Mix (Invitrogen^TM^, Thermo Fisher Scientific, Inc., Waltham, MA, USA), and subsequently with proteinase K (20 mg mL^−1^) for 2 h at 37 °C. The solution was treated with 100 μL of 1.5 M NaCl, and DNA was extracted with phenol/chloroform. A total of 0.1 volumes of 3 M sodium acetate pH 5.2 and 2.5 volumes of cold ethanol were used for DNA precipitation. The DNA was then pelleted, washed with 70% ethanol, resuspended in 30 μL of molecular-biology grade water (Sigma-Aldrich, Saint Louis, MO, USA), and stored at −20 °C until use. DNA concentration and quality were determined in a BioTek Epoch™ spectrophotometer system (Thermo Fisher Scientific, Inc., Waltham, MA, USA). DNA extraction yields from H-07, H-12 and H-13 samples selected for fosmid library preparation were comprised between 15 and 40 ng mL^−1^. Bacterial load in bile samples was above 10^2^–10^3^ cells mL^−1^ in all samples. Bile microbiota is characterised by low microbial biomass although these bacterial communities are biologically relevant according to previous work [1]. Fosmid libraries were obtained using the CopyControl- Fosmid Library Production Kit (Epicentre^®^ Biotechnologies, and Illumina^®^ Company, San Diego, CA, USA) following the manufacturer’s instructions.

#### 2.2.2. High-Molecular-Weight DNA Purification and Fractionation

The extracted high-molecular-weight (HMW) DNA was end-repaired to generate blunt-ended 5′-phosphorylated DNA, and then size-separated on a 1% low-melting-point agarose gel (Sigma-Aldrich, Saint Louis, MO, USA) using pulse-field gel electrophoresis (PFGE) (BIO-RAD Laboratories Inc. Hercules, CA, USA) at 35 V cm^−1^ for 15 h. In addition to the sample DNA, each gel contained a High Range DNA Molecular marker (Gen Ruler, Thermo Fisher Scientific, Inc., Waltham, MA, USA), and a fosmid control DNA (Epicentre^®^ Biotechnologies, and Illumina^®^ company, CA, USA). The sample DNA was cut out for a size suitable for fosmid cloning (~40 kb) and extracted with GELase (Epicentre^®^ Biotechnologies, and Illumina^®^ company, CA, USA) following the manufacturer’s protocol.

#### 2.2.3. Library Construction and Isolation

A total of 20 μg of size-separated DNA of ~40 kb were cloned into the copy control pCC1FOS vector following the kit’s protocol. This vector allows cloning large DNA inserts into a stable low-copy-number vector, and thus the library could be screened not only for small genes but also for whole operons. After cloning, the pCC1FOS vector containing the DNA inserts were packaged into the MaxPlax Lambda Phage (Epicentre^®^ Biotechnologies, and Illumina^®^ company, CA, USA), and an overnight culture of *Escherichia coli* EPI300-T1^R^ (Supplied by Epicentre^®^ Biotechnologies, and Illumina^®^ Company, CA, USA) grown in Luria–Bertani (LB) media supplemented with 10 mM MgSO4 + 0.2% Maltose was incubated with the packaged fosmids during 1 h at 37 °C to infect the EPI300-T1^R^ cells. Due to the low amount of bacterial DNA on bile samples, and to avoid fosmids that had inserted DNA belonging to *E. coli*, the library was subjected to a thermal shock (70 °C) and then growth 4 h on LB broth supplemented with 12.5 μg mL^−1^ chloramphenicol, being obtained a pool of fosmid clones instead of isolated clones, with an average size of the inserts of 25 kbp, Fosmid library was then stored at −80 °C until analysis.

The sequencing of the fosmid pool was carried out in Genprobio s.r.l. (Parma, Italy (http://www.genprobio.com/ last accessed: 30 September 2021). Library construction was performed according to Genprobio standard protocols and sequencing of the fosmid library was carried out in a NextSeq platform from Illumina. Reads belonging to the fosmid pCC1FOS were removed and the resulting six and a half paired-ends (2 × 150 bp) million reads were assembled in contigs.

### 2.3. Functional Analysis of Bile Metagenome: Antibiotic and Multidrug Resistance Genes (ARGs and MDRs)

The presence of several ARGs in bile metagenome, as well as fosmid library samples, was determined using TORMES pipeline v.1.2.1 [13], with default options. It should be noted that this pipeline implements all analysis steps required to process raw sequencing data. Fosmid library sample had been previously assembled into contigs by the sequencing service. Bile metagenomes and fosmid library were mapped against Resfinder [14], and the Comprehensive Antibiotic Resistance Database (CARD) [15], databases to identify mainly ARGs although some MDRs can be also identified using the TORMES pipeline.

To deepen the MDR study, an additional analysis based on MDR annotation using the Pfam database was performed. For this purpose, sequences were assembled and total coding sequences were predicted using FragGeneScan v.1.31 [16]. Then, the segmasker software v.2.2.28 was used to identify repetitive regions in putative open reading frames, and coding sequences containing over 40% repetitive sequence were removed. Filtered coding sequences were annotated using HMMER software v.3.3 for biosequence analysis using profile hidden Markov models (HMMs) and Pfam database [17,18]. Once protein functional domains were annotated, those having Pfam entries that may correspond to bacterial MDRs described in the Transporter Classification Database (TCDB) database [19], were selected for comparative analysis. Furthermore, the distribution of these MDR functional domains in bile samples was compared to the one observed in 40 intestinal metagenomes sequenced by Kovatcheva-Datchary et al. [20], which were pre-assembled in a previous work [21], and were introduced as inputs in this pipeline.

**Figure 1:**
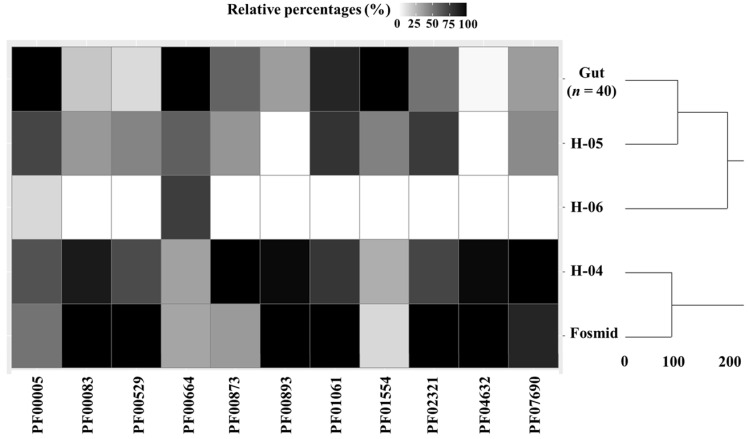
Heatmap showing the abundance of multidrug resistance genes (MDRs) in bile metagenome samples H-04, H-05 and H-06, a fosmid library and gut metagenome samples used as control. These abundances were expressed as relative percentages (%). MDR are annotated according to reference codes retrieved from the Pfam database. Similarities between samples are also illustrated in a dendrogram and expressed as distances between their characteristic MDRs profiles calculated by the complete linkage method (vertical axis).

To compare MDRs distribution in different samples under study, the number of each MDR domain per sequence length (in base pairs) was first calculated. Then, these values were transformed to relative percentages: abundances of individual MDRs in each sample were multiplied by 100 and divided by maximum abundance values observed for that MDR domain (i.e., those values corresponding to the sample showing the highest abundance of that MDR domain). In this sense, relative percentages highlight differences existing between samples and can be useful to determine MDR enrichment in bile microbiota compared to gut microbiota used as control (Figure 1). Then, sequences were clustered to determine similarities in their characteristic MDRs profiles. These clusters were calculated by the complete linkage method. In the complete linkage method, all pairwise dissimilarities between the elements in each cluster (i.e., metagenomes and fosmid library samples) are calculated using the basic function “hclust” from R v.3.6.2 programming environment. Then, the largest dissimilarity value is chosen as the distance between clusters. Therefore, this method produces more compact clusters.

### 2.4. Functional Analysis of Bile Metagenome: Metagenomic Dark Matter

Remote homology analysis to annotate metagenomic dark matter sequences found in biliary metagenomes and fosmid library through profile-profile comparisons was computed as previously described by Lobb et al. [9]. Briefly, quality filtered metagenomic reads obtained by TORMES pipeline v.1.2.1 [13], were assembled using a combination of megahit v.1.1.3 and metaspades v.3.13.0 software implemented in metaWRAP v.1.3.2 pipeline [22], to enhance the quality of assemblies. It should be noted that the fosmid library sample was already assembled by the sequencing service. Then, total coding sequences were predicted in assembled metagenomes and fosmid library using FragGeneScan v.1.31 [16], and segmasker software v.2.2.28 was used to remove coding sequences containing over 40% repetitive sequence. Filtered coding sequences were annotated using HMMER software v.3.3 for biosequence analysis using profile hidden Markov models (HMMs) and Pfam database [17,18], as well as rpsblast software from BLAST suite v.2.10.1+ and the Conserved Domain Database (CDD) [23]. An *E*-value cut-off of 10^−3^ was considered for both methods. Unidentified coding sequences were clustered with CD-HIT v.4.8.1 [24], using a 65% identity threshold. Singleton clusters were removed as well as clusters whose longest sequence was shorter than 100 amino acids and the clusters comprised entirely of sequences with 99% or greater identity to the representative sequence. MUSCLE software v. 3.8.1551 [25], was used to generate multiple sequence alignments of remaining clusters that were enlarged with sequences from the *UniProt20* database using HHblits v.3.3.0 [26]. Finally, profile-profile comparisons were performed using HHsearch v.3.3.0 and PDB70 database [26]. This pipeline was also run using shuffled cluster sequences to avoid false-positive prediction. The presence of annotated bile dark matter functional domains in faecal metagenomes obtained by Kovatcheva-Datchary et al. [20], and processed in the previous section of this work, was also investigated.

A complementary analysis of protein functional domains identified in bile metagenomic dark matter, following the protocol above described, was performed using molecular modelling techniques. Specifically, potential mechanisms of action of putative 1D-myo-inositol 2-acetamido-2-deoxy-α-D-glucopyranoside deacetylase annotated in the fosmid library were elucidated. With this aim, the possible 3D conformation of this functional domain was generated via homology modelling [27], and active site prediction [28]. The structure of its main substrate (1D-myo-inositol 2-acetamido-2-deoxy-α-D-glucopyranoside) was retrieved from the ChemSpider database (http://www.chemspider.com/ last accessed: 30 September 2021). Water molecules and heteroatoms were removed from all structural models (in pdb format) and energy was minimised using Swiss-PdbViewer (http://www.expasy.org/spdbv/ last accessed: 30 September 2021).

Potential interaction mechanisms that may take place in the active site of this functional domain were first studied by molecular docking. In this sense, grid boxes (15 × 15 × 15 Å) were placed in the annotated active site and molecular docking simulations of the metagenomic dark matter functional domain (putative 1D-myo-inositol 2-acetamido-2-deoxy-α-D-glucopyranoside deacetylase) and its substrate (1D-myo-inositol 2-acetamido-2-deoxy-α-D-glucopyranoside) were performed on Autodock Vina [29]. Calculation of Root Mean Squared Deviation (RMSD) of docking results was performed using the LigRMSD server (https://ligrmsd.appsbio.utalca.cl/ last accessed: 30 September 2021). In addition, a molecular dynamics simulation of the enzyme-substrate complex was run to calculate enzyme-substrate binding energies by MM/PBSA method [30], implemented in GROMACS [31]. Input files for molecular dynamics simulation were generated using CHARMM-GUI (http://www.charmm-gui.org/ last accessed: 30 September 2021). CHARMM36m forcefield was selected (http://mackerell.umaryland.edu/charmm_ff.shtml last accessed: 30 September 2021). Molecular dynamics simulations were carried out in explicit solvent following the standard procedure indicated by CHARMM-GUI. The solvent temperature was 37 °C and salt concentration, expressed as NaCl ions, was set at 0.149 M according to bile sample metadata [1]. Simulations were equilibrated for 125 ps and run for 1 ns. Snapshots were taken every fifty molecular dynamics simulation steps.

The computational workflow here described was also used to analyse faecal metagenome samples obtained by Kovatcheva-Datchary et al. [20].

## 3. Results and Discussion

Human bile metagenomes obtained from the gallbladder have been characterised with an emphasis on proteins conferring antibiotic resistance and multidrug resistance proteins, as well as several protein functional domains with unknown function, comprising so-called metagenomic dark matter. For this purpose, a comprehensive data analysis strategy involving two different steps was followed: (1) identification of ARGs and MDRs and (2) tentative assignment of biological functions to metagenomic dark matter domains through remote homology and computational chemistry techniques.

Biliary fosmid libraries as well as shotgun metagenomic sequences obtained in a previous work [1], were selected to perform this study. Taxonomic identification of fosmid library using TORMES software [13], revealed five major phyla: Firmicutes, Proteobacteria, Bacteroidetes, Epsilonproteobacteria and Actinobacteria. These phyla were also the most abundant according to 16S sequencing experiments of bile samples performed in a previous study [1]. Main families present in fosmid library include Caulobacteraceae, Chitinophagaceae, Lachnospiraceae, Gammaproteobacteria, Sphingomonadaceae, Ruminococcaceae, Muribaculaceae, Rikenellaceae, Burkholderiaceae, Bacteroidaceae, Streptomycetaceae, Enterobacteriaceae, Barnesiellaceae and Prevotellaceae in agreement with 16S sequencing data of bile samples used to prepare fosmid library reported by Molinero et al. [1]. Similarly, the main genera found in the fosmid library include *Sphingomonas, Alistipes, Bacteroides, Streptomyces, Barnesiella*, *Prevotella, Alloprevotella, Lachnospira* and an unclassified genus from the Muribaculaceae family, which were also identified by 16S sequencing of these bile samples [1]. Therefore, 16S sequencing and fosmid library preparation can be used to identify similar bacterial groups corresponding to the most abundant taxa in bile microbiota. These results indicate that the fosmid library allows capturing major bacterial populations present in bile microbiota in agreement with previous studies available in the literature [1,2].

### 3.1. Functional Analysis of Bile Metagenome: Antibiotic and Multidrug Resistance Genes

The presence of genes presumptively conferring antibiotic resistance ARGs was determined (Supplementary Materials Table S1). The sequences of the fosmid library were included in subsequent analyses. It has been demonstrated that bacterial resistance to several antibiotics like ampicillin and tetracycline may be related to the presence of bile salts [32]. In this sense, reference databases containing ARGs and MDRs and proteins have been developed including Resfinder [14], CARD [15], and TCDB [19]. Moreover, computational pipelines to automatically determine ARGs are currently available [13].

With regard to metagenome samples, H-04 showed ARGs conferring resistance to the widest range of antibiotics: amoxicillin, ampicillin, cephalosporin, cephalothin, chloramphenicol, doxycycline, lincosamide, macrolide, minocycline, monobactam, penam, penem, piperacillin, streptogramin, tetracycline, tetracycline and ticarcillin. H-05 showed ARGs conferring resistance to doxycycline, minocycline, tetracycline as well as aminoglycoside. In contrast, only one ARG conferring resistance to chloramphenicol was found in the H-06 sample, probably due to its lower number of reads and a high percentage of unassigned sequences as previously reported by Molinero et al. [1]. However, the greatest variety of ARGs was found in the fosmid library although ARGs abundances, expressed as the number of ARGs conferring resistance to each type of antibiotic per sequence megabase, were in the range of those observed in bile metagenome samples (Supplementary Materials Table S1). These domains included ARGs conferring resistance to aminocoumarin, aminoglycoside, azithromycin, carbapenem, cephalosporin, cephamycin, chloramphenicol, diaminopyrimidine, erythromycin, fluoroquinolone, fosfomycin, glycylcycline, lincosamide, macrolide, monobactam, nitroimidazole, oxazolidinone, penam, penem, phenicol, pleuromutilin, rifamycin, streptogramin, tetracycline, triclosan and trimethoprim. Previous studies revealed that the resistance of *Salmonella typhimurium* to ampicillin and tetracycline was increased in the presence of bile salts [32], and several ARGs conferring resistance to these two antibiotics were also found in H-04, H-05 biliary metagenomes and that of sequences derived from fosmid clones, suggesting that bile could select for these specific resistances. Interestingly, several proteins involved in multidrug resistance were exclusively found in the fosmid library including GadW and GadX (two AraC-family regulators that promote mdtEF expression to confer multidrug resistance), multidrug resistance protein MdtH and multidrug resistance lipid transporter MsbA, which is a bacterial ABC transporter that is essential for cell viability. Additional MDR domains were annotated using a complementary analysis based on Pfam [17,18], and TCDB databases [19]. According to the results obtained, several MDRs were more abundant in the fosmid library and the bile metagenome from sample H-04 than in control gut metagenomes sequenced by Kovatcheva-Datchary et al. [20] (Figure 1): (1) PF00083 multidrug resistance efflux pump from Actinobacteria that exports fluoroquinolones and chloramphenicol, (2) PF00529 multidrug-resistant pump constituent from Proteobacteria, (3) PF00873 multidrug efflux pump from Proteobacteria, (4) PF00893 multidrug efflux pump from Proteobacteria that takes cationic lipophilic drugs as substrate, (5) PF02321 resistance efflux pump complex from Proteobacteria, (6) PF04632 multidrug resistance protein from Proteobacteria, (7) PF07690 multidrug resistance efflux transporter from Actinobacteria and Proteobacteria. In contrast, other domains were more abundant in gut metagenome (Figure 1): (1) PF00005 multidrug resistance exporter from Actinobacteria, (2) PF00664 multidrug efflux pump from Actinobacteria, (3) PF01554 multidrug-resistance efflux pump from Proteobacteria. No differences in the abundance of PF01061 multidrug resistance exporter from Actinobacteria were found between bile and faecal metagenomes sequenced by Kovatcheva-Datchary et al. [20], used as control (Figure 1). In general, bile samples showed greater abundances of MDRs compared to gut metagenomes. It has been reported that several bacteria develop multidrug resistance strategies to resist the bactericidal activity of bile [33]. This fact may be attributed to major bile components, such as bile acids and salts, cholesterol, and bilirubin [1], that may react with free radicals contributing to oxidative stress in the gallbladder ecosystem [34]. Previous studies highlight that oxidative stress derived from bile salt exposure stimulates antibiotic resistance mechanisms and MDRs expression [32,35]. These mechanisms allow bacteria to survive in certain ecological niches alongside bile salts. In this regard, some MDR transporters display a dual activity, being able to transport bile salts and antibiotics [36]. It has been reported that bacteria oxidative stress promotes horizontal transfer of multidrug resistance genes within and across bacterial genera as a defensive response [37]. Multidrug proteins identified in this work corresponded to Actinobacteria and Proteobacteria, two of the most important phyla from gallbladder microbiota [1]. Previous studies comparing metabolic capabilities of gallbladder and gut microbiota suggest that gallbladder microbiota shows higher abundances of genes involved in bile acid metabolism as well as bile and multidrug resistance [1,3]. Therefore, it is reasonable to assume that bile microbiota is subjected to higher oxidative stress than gut microbiota, leading to higher abundances of MDRs.

The identification of several ARGs and MDRs in the bile metagenome indicated that gallbladder microbiota constitutes a reservoir of antibiotic resistant bacteria. In this sense, bile is one innately bactericidal compound present in humans and plays a major role in reducing the bacterial burden in the gastrointestinal tract and aiding in digestion [33]. Bile release to the intestinal lumen is an essential step to gastrointestinal transit and gut pathogens may be exposed to antibiotic resistant-bacteria present in bile. Pathogen interactions with biliary microbiota may lead to horizontal gene transfer allowing bacteria to exchange their genetic materials, including ARGs and MDRs. As a consequence, gut pathogens may develop new resistance mechanisms to antibiotics and other bactericidal components and use bile as a localisation signal to regulate virulence gene expression and enhance infection [35,38]. Therefore, the presence of ARGs and MDRs in gallbladder microbiota may have relevant implications in human health and is necessary to understand these antimicrobial resistance mechanisms to develop successful therapeutics and clinical interventions [33].

### 3.2. Functional Analysis of Bile Metagenome: Metagenomic Dark Matter

Functional analysis of the bile metagenome revealed the presence of several ARGs and MDRs. However, the biological functions of a wide range of protein functional domains found in metagenomic reads remain unknown. To deepen the study of these sequences and to propose potential activities for these novel domains, remote homology techniques were employed as previously suggested [9]. According to the results obtained, a total of 8 protein-coding sequence clusters with no domain family homologs when mapped against reference databases, showed high probabilities (above 90%) to exert certain activities during profile-profile comparisons using remote homology techniques (Table 1). Among these novel functional domains, two enzymes involving acetyltransferase and 1D-myo-inositol 2-acetamido-2-deoxy-α-D-glucopyranoside deacetylase activities were found. In addition, other unspecific domains such as two outer membrane transport, including an iron transporter protein, an outer membrane assembly factor, a DNA repair and recombination protein and a response regulator phosphatase were identified. One two-domain protein structure was also determined although its specific function could not be fully elucidated by remote homology. The distribution of these functional domains in bile samples was compared to the one observed in 40 faecal metagenomes from healthy individuals [20], which were pre-assembled in a previous study [21]. As it can be observed in Table 1, most metagenomic dark matter functional domains were identified in sequence from the H-04 sample and the fosmid library, while no functional domains could be determined in H-06 probably due in part to the low number of filtered reads obtained from this sample and the higher percentage of previously assigned sequences [1]. In contrast, H-05 showed a similar pattern to gut metagenomes, where only one outer membrane protein assembly factor was identified.

Interestingly, putative 1D-myo-inositol 2-acetamido-2-deoxy-α-D-glucopyranoside deacetylase found in the fosmid library catalyses the hydrolysis of 1D-myo-inositol 2-acetamido-2-deoxy-α-D-glucopyranoside to form 1D-myo-inositol 2-amino-2-deoxy-α-D-glucopyranoside and acetate, the fourth overall step in mycothiol biosynthesis [39]. Mycothiol is the major low-molecular-weight thiol in Actinobacteria and is critical for the survival of this phylum subjected to oxidative stress by protecting cells against reactive oxygen species and reactive nitrogen intermediates [39,40]. As previously explained, Actinobacteria was one of the main phyla identified in the bile metagenome in agreement with other studies, comprising 3–13% gallbladder microbiota [1]. Furthermore, it has been demonstrated that mycothiol plays a key role in the protection of cells against toxic compounds such as antibiotics [41]. This fact may explain the presence of putative enzymes involved in mycothiol synthesis in the fosmid library, the sample showing the widest range of ARGs and MDRs. Presumably, the fosmid library was generated using samples subjected to the greatest oxidative stress. It should be noted that mycothiol contributes to the detoxification of reactive oxygen species and its levels vary in response to changes in growth conditions such as oxidative stress. Therefore, genes involved in mycothiol biosynthesis may be upregulated under these conditions [42]. On the other hand, H-04 showed the widest range of membrane proteins in its metagenomic dark matter fraction.

Functional analysis of gallbladder microbiota performed by Molinero et al. [1], revealed the presence of clusters of orthologous groups associated to some of the metabolic functions above mentioned, including replication, recombination and repair domains, signal transduction mechanisms, extracellular structures and inorganic ion transporters. In this sense, in agreement with our results, it has been reported that biliary microbiota is enriched in membrane transporters compared to faecal microbiota [3]. On the other hand, in the study of Song et al. [2], a higher proportion of deacetylases and amino acid transporters was observed in bile samples from gallbladder cancer patients, highlighting the potential role of deacetylases, which were also present in the metagenomic dark matter fraction. The largest functional domain cluster reported by Molinero et al. involved bile metagenomic sequences with unknown functions (comprising 24.5–30.8% of total sequences) [1]. It should be taken into account that scarce information is available on bile metagenome due to difficulties in accessing solid biopsies or liquid fluids and low bacterial load, leading to a larger number of unclassified sequences when compared to relatively well-known human ecosystems such as gut microbiota. As a consequence, new methodologies to annotate unidentified sequences could be of great interest. In the present work, bile-specific protein functional domains have been identified in the metagenomic dark matter fraction, including enzymes involved in the biosynthesis of protective compounds that may play a key role on bacterial resistance to bile exposure.

Shotgun metagenomics sequencing and fosmid library preparation are two complementary approaches to study gallbladder microbiota. Fosmid library offers no limitations in DNA concentration. However, shotgun metagenomics requires minimal high-quality DNA concentrations isolated from biological samples that may be difficult to handle such as bile samples. This advantage can be of special interest to characterise low bacterial biomass ecological niches like gallbladder. In addition, fosmid libraries allow studying many complete genes that are not fully captured in metagenomic libraries. On the other hand, shotgun metagenomics is less labour-intensive, yields sequencing data faster than fosmid libraries and avoids common biases introduced through PCR- or activity-guided functional metagenomics workflows [43]. In the present study, the use of fosmid libraries allowed the identification of novel acetyltransferase and deacetylase enzymes from the microbial dark matter fraction. These novel functional domains could not be captured by shotgun metagenomic sequencing and highlight the suitability of fosmid library preparation for the functional characterisation of a scarcely ecological niche like bile microbiota.

#### Molecular Modelling Techniques to Study Biological Functions of Metagenomic Dark Matter

**Figure 2:**
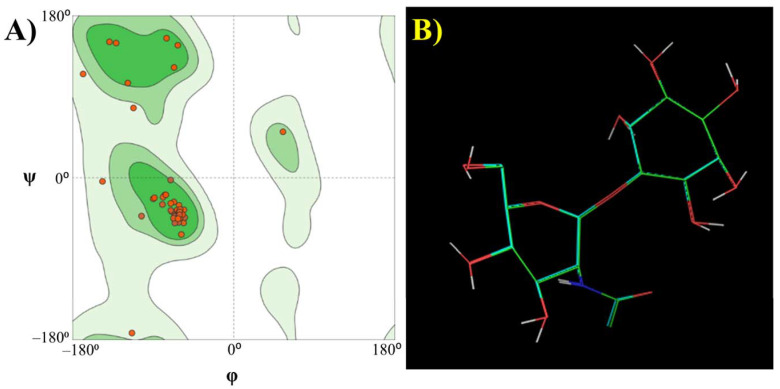
(**A**) Ramachandran plots showing the values of phi (φ) and psi (ψ) angles of 3D structures of putative 1D-myo-inositol 2-acetamido-2-deoxy-α-glucopyranoside deacetylase generated by homology modelling. Dots represent Φ and ψ angles of individual amino acid residues contained in the enzyme structure. Coloured regions of the plot indicate the allowed regions corresponding to favoured (green) and allowed (light green) combinations of torsional angles are possible. As it can be observed, no amino acid residues can be found in the disallowed region (white), indicating the suitability of the structural model to perform further analyses. (**B**) Redocking results of 1D-myo-inositol 2-acetamido-2-deoxy-α-glucopyranoside to putative 1D-myo-inositol 2-acetamido-2-deoxy-α-glucopyranoside deacetylase. The similarity between the docked and bound conformations of the substrate in the protein structure is illustrated. Colour codes are assigned to different atoms present in 1D-myo-inositol 2-acetamido-2-deoxy-α-glucopyranoside: green (carbon), hydrogen (white), oxygen (red) and nitrogen (blue). It should be noted that this Figure illustrates the superimposition of two structures (docked and redocked). Superimposed docked and redocked conformation are very similar, highlighting the reproducibility of molecular docking simulations.

Putative functions of some protein functional domains present in the dark matter fraction of bile metagenomes have been annotated by profile-profile comparisons by remote homology techniques. However, these methods still depend on sequence comparison and mechanisms of action of these domains involved in biological processes might be difficult to confirm. To deepen the characterisation of potential enzymes found among unidentified reads, we propose a complementary analysis of metagenomic dark matter sequences based on molecular modelling techniques. Among protein functional domains annotated in the previous step, putative 1D-myo-inositol 2-acetamido-2-deoxy-α-D-glucopyranoside deacetylase could be the most biologically relevant one. Therefore, potential mechanisms of action of this functional domain present in the fosmid library have been elucidated by a computational chemistry approach. For this purpose, a possible 3D structure of this functional domain sequence was first generated by homology modelling [27]. The quality of the 3D structure generated was assessed through the Ramachandran plot (Figure 2A). All amino acid residues were present in the allowed region of the conformational space and no Ramachandran outliers were observed. Moreover, quality indicators Q-Mean and Z-score were −0.8 and 1.9, highlighting the suitability of this model to perform the simulations.

Interaction mechanisms between this enzyme functional domain and its corresponding substrate (1D-myo-inositol 2-acetamido-2-deoxy-α-D-glucopyranoside) were investigated through molecular docking. According to the results obtained, the mean affinity values of the top nine positions given by Autodock Vina was −3.82 ± 0.22 kcal mol^−1^. Negative values indicate that substrate binding to the active site of the putative enzyme is a thermodynamically favoured reaction as expected.

**Figure 3:**
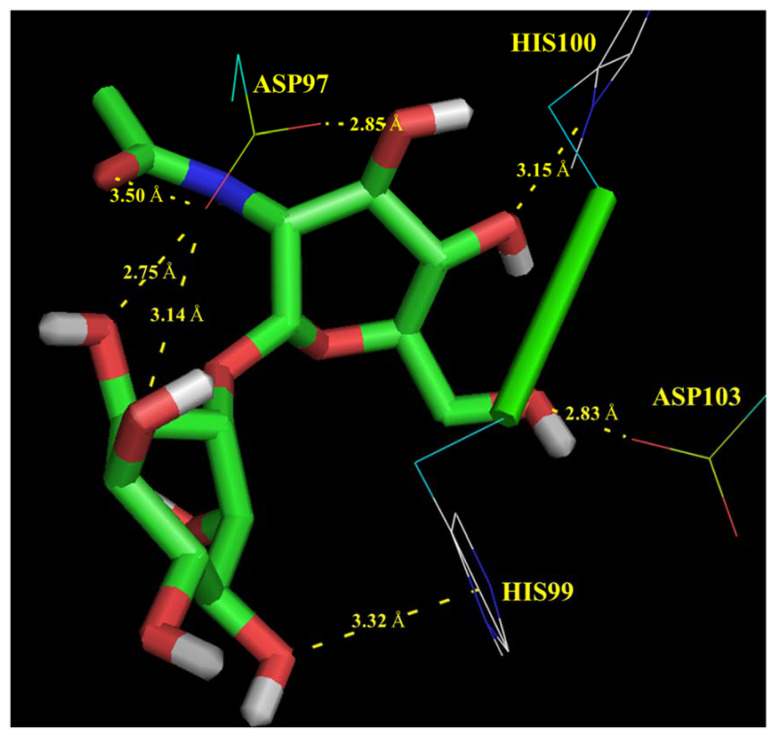
Potential interaction mechanisms, determined by molecular docking, between 1D-myo-inositol 2-acetamido-2-deoxy-α-glucopyranoside and putative 1D-myo-inositol 2-acetamido-2-deoxy-α-glucopyranoside deacetylase annotated in the metagenomic dark matter fraction of fosmid library. Polar contacts between the substrate and aspartic acid (ASP) and histidine (HIS) residues from the active site are highlighted in yellow. Bond distance is expressed in Angstroms (Å).

Molecular docking simulations revealed several chemical interactions between the putative enzyme and its corresponding substrate, consisting mainly of polar contacts between aspartic acid (ASP) or histidine (HIS) catalytic residues from the active site, and hydroxyl or acetamido groups from the substrate. These interaction mechanisms are illustrated in Figure 3 and discussed below:

Residue ASP97 interacts with oxygen atoms from hydroxyl and acetamido groups of 2-acetamido-2-deoxy-α-D-glucopyranoside monomer showing bond lengths of 2.85 and 3.50 Å, respectively.

Residue ASP97 interacts with oxygen atoms from hydroxyl groups of 1D-myo-inositol showing bond lengths of 2.75–3.14 Å.

Residue HIS99 interacts with oxygen atoms from hydroxyl groups of 1D-myo-inositol monomer showing a bond length of 3.32 Å.

Residues HIS100 and ASP103 interact with oxygen atoms from hydroxyl groups of 2-acetamido-2-deoxy-α-D-glucopyranoside monomer showing bond lengths of 3.15 and 2.83 Å, respectively.

To assess the reproducibility of molecular docking simulations and to ensure the quality of our results, the substrate was redocked to the active site of the putative enzyme in replicate (*n* = 10). Docked and bound conformations of the substrate were compared, showing a high degree of similarity assessed through the superimposition of both structures (Figure 2B) and low values of RMSD (0.097 ± 0.064 Å).

Interaction mechanisms here described are similar to those reported by several authors for similar enzymes using experimental methods as well as in silico techniques like molecular docking and molecular dynamics simulations [39,40,44,45,46,47]. These authors also highlighted the catalytic function of ASP and HIS residues. It has been described that these residues form hydrogen bonds, hydroxyl groups, from the substrate which may also serve to mediate binding of the substrate and to orient the glucosamine in a conformation suitable for catalysis [44,46,47], in agreement with our results.

**Figure 4:**
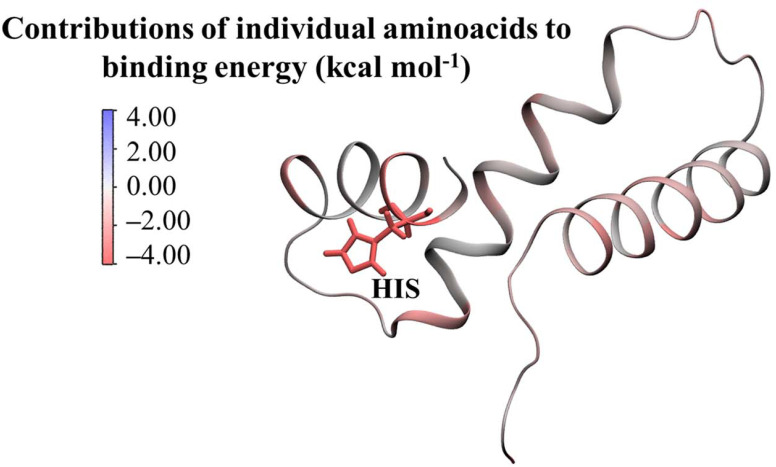
Individual contributions of each amino acid residue from putative 1D-myo-inositol 2-acetamido-2-deoxy-α-glucopyranoside deacetylase annotated in the metagenomic dark matter fraction of fosmid library to total binding energy in the enzyme-substrate complex. The greatest individual energy contributions were observed for histidine residues (HIS) present in the active site of the putative enzyme.

Affinity values here described were lower than the binding energies obtained previously by molecular docking of well-characterised bacterial 1D-myo-inositol 2-acetamido-2-deoxy-α-D-glucopyranoside deacetylases to their substrates [44]. However, few studies reported binding energies of these complexes while molecular docking results are used to roughly estimate the relative binding affinities of compounds to a target and do not usually correlate well with the real values of binding energies. To overcome this limitation, molecular dynamics simulation, a more advanced technique, was employed to calculate more accurate binding energies. According to the results obtained, the binding energy enzyme-substrate complex determined by MM/PBSA method was −10.97 ± 0.08 kcal mol^−1^, which was in the range of binding energies reported by Huang et al. for similar enzymes using molecular modelling techniques [44]. Individual contributions of each amino acid residue to total binding energy were calculated and are illustrated in Figure 4. As expected, those amino acids present in the active site (involving mainly ASP and HIS residues) showed negative energies (highlighting the binding mode of the substrate to this region). It has been reported that HIS mediate pyranose binding while ASP act as catalytic acid in this type of enzyme [47]. This fact may explain the highest energy contribution observed for HIS residues, playing a key role in substrate binding to the active site.

**Figure 5:**
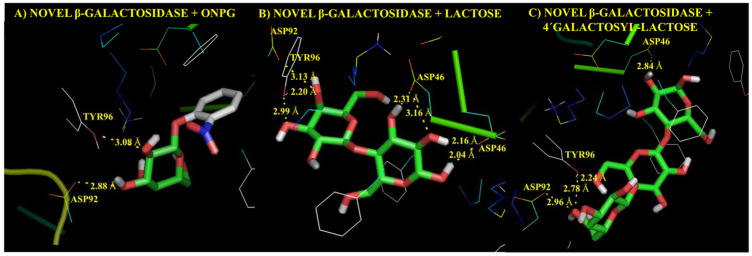
Potential interaction mechanisms, determined by molecular docking, between novel β-galactosidase 2 annotated in the metagenomic dark matter fraction of gut metagenomes and several substrates: (**A**) ortho-nitrophenyl glucoside (ONPG), (**B**) lactose, (**C**) 4′galactosyl-lactose. Potential contacts between hydroxyl groups of substrates, and aspartic acid (ASP) and tyrosine (TYR) residues from the active site are highlighted in yellow. Bond distance is expressed in Angstroms (Å).

Molecular modelling of putative 1D-myo-inositol 2-acetamido-2-deoxy-α-D-glucopyranoside deacetylase found in the dark matter fraction of bile metagenome has been used to confirm the potential enzymatic activity suggested by remote homology profile-profile comparisons. This novel approach may be of special interest to discover novel microbial enzymes which had not been previously characterised. To illustrate potential future applications of this method, more complex and more abundant metagenomes such as faecal metagenomes were also analysed following the computational workflow here described. For this purpose, faecal metagenomes obtained by Kovatcheva-Datchary et al. [20], were selected. This dataset comprising 40 gut metagenomes from healthy individuals was used as a control in the previous data analysis steps (Figure 1, Table 1). Computational elucidation of the metagenomic dark matter fraction found in gut metagenomes revealed the presence of up to 1509 novel protein-coding sequences. Among these sequences, biological functions of 792 protein-coding sequences were annotated by remote homology. In addition, novel carbohydrate-active enzymes were annotated, including three β-galactosidase domains (novel β-galactosidases 1, 2 and 3). Novel β-galactosidases 1, 2 and 3 were present in 90.0, 42.5 and 45.0% of gut metagenomes under study. Interestingly, remote homology analysis suggested that novel β-galactosidases 2 belongs to *Roseburia hominis*, an emerging potentially beneficial bacterium that may have a positive influence on human health [48]. Then, 3D structural models of novel β-galactosidases were generated by homology modelling (an example of a novel β-galactosidase structure is provided in Supplementary Materials Figure S1). Molecular modelling was performed to investigate interaction mechanisms between novel β-galactosidases and three common substrates for β-galactosidases retrieved from PubChem (https://pubchem.ncbi.nlm.nih.gov/ last accessed: 30 September 2021): ortho-nitrophenyl glucoside (ONPG), lactose and 4′galactosyl-lactose (a representative galacto-oligosaccharide structure) leading to affinity values of −6.0, −5.7 and −6.0 kcal mol^−1^, respectively. As explained, negative affinity values indicate that substrate binding to the active site of the putative β-galactosidase is a thermodynamically favoured reaction. Enzyme-substrate complexes were redocked to assess the reproducibility of the method. As expected, docked and bound conformations of substrates showed a high degree of similarity (Supplementary Materials Figure S2). Potential chemical interactions between novel β-galactosidases and these substrates are illustrated in Figure 5. Chemical interactions between hydroxyl groups of substrates and aspartic acid (ASP) and tyrosine (TYR) residues from the active site of β-galactosidases were found. These interactions agree with characteristic mechanisms of action of β-galactosidases reported by previous authors in molecular modelling studies [49,50]. Therefore, novel β-galactosidases from health-promoting gut commensals have been identified in the metagenomic dark matter fraction of gut microbiota using a combination of remote homology and molecular modelling techniques.

Few studies developed specific computational pipelines and software packages to analyse the microbial dark matter fraction resulting from current metagenomic sequencing techniques. Alternative approaches developed by previous authors involved phylogenetic neighbour analysis of sequences to assess how well microbial communities are represented by the available completed genomes [5], and network analysis of unidentified bacteria to investigate their role in complex microbial ecosystems from all environments [8]. However, these methods do not provide an exhaustive annotation of potential biological functions of protein domains present in the metagenomic dark matter fraction. In this sense, applications of remote homology to annotate unidentified coding sequences have been reported [9]. On the other hand, recent studies suggest that molecular modelling is a valuable tool to elucidate potential mechanisms of action of bacterial enzymes [12]. Therefore, the combination of both techniques (remote homology and molecular modelling) can be used to annotate novel protein sequences and to ensure these sequences show the characteristic mechanisms of action associated with that specific type of protein. To our knowledge, no existing software allows an exhaustive annotation of novel bacterial enzymes and their chemical mechanisms of action. In the present work, a novel deacetylase domain was annotated by remote homology. Then, molecular modelling of this domain revealed a deacetylase-like behaviour based on potential enzyme-substrate interactions. To experimentally validate the computational method here presented, future studies will cover recombinant protein expression in bacteria and functional-structural analyses of novel enzymes. Structural conformations of enzymes will be compared to in silico models presented in this work to refine computational predictions.

## 4. Conclusions

The present work provides a comprehensive characterisation of the biliary microbiome with an emphasis on ARGs, MDRs and metagenomic dark matter fraction. ARGs and MDRs were identified, showing the highest abundances in a fosmid metagenome library. Several protein functional domains have been annotated in the metagenomic dark matter fraction including acetyltransferases, outer membrane transporter proteins, membrane assembly factors, DNA repair and recombination proteins and response regulator phosphatases. In addition, a novel bacterial deacetylase involved in mycothiol biosynthesis and may exert a protective effect on Actinobacteria has been annotated in the metagenomic dark matter fraction of the fosmid library. Potential mechanisms of action of this novel deacetylase have been elucidated by molecular docking and molecular dynamics simulations, highlighting the role of histidine and aspartic acid residues from the active site. Besides, the binding energy of the enzyme-substrate complex has been estimated (−10.97 ± 0.08 kcal mol^−1^). This integrated approach allows an exhaustive characterisation of multiple genes and enzymes involved in bacterial resistance against bile components. The combination of remote homology and molecular modelling techniques provides a deeper characterisation of functional domains present in metagenomic dark matter and could be used in future studies for novel enzyme discovery.

## Figures and Tables

**Table 1:** Identification of several functional domains annotated in the metagenomic dark matter fraction of bile metagenome samples H-04, H-05 and H-06, a fosmid library and gut metagenome samples used as control, using remote homology techniques.

Dark Matter Functional Domain	Number of Samples Containing Each Domain / Total Number of Samples				
	H-06 (*n* = 1)	H-05 (*n* = 1)	H-04 (*n* = 1)	Fosmid (*n* = 1)	Gut Metagenome (*n* = 40)
Acetyltransferase	-	-	-	1/1	-
Deacetylase	-	-	-	1/1	-
RNA repair and recombination protein	-	-	1/1	-	-
Outer membrane protein	-	-	1/1	-	-
Outer membrane ion transport	-	-	1/1	-	-
Outer membrane protein assembly factor	-	1/1	-	-	34/40
Response regulator aspartate phosphatase	-	-	1/1	-	-
Uncharacterised two-domain protein structure	-	-	1/1	-	1/40

## Data Availability

Fosmid library contigs in FASTA format are publicly available at the Sequence Read Archive (SRA) of the National Center for Biotechnology Information (NCBI) (https://www.ncbi.nlm.nih.gov/bioproject/PRJNA769070/). Data were deposited under the BioProject number PRJNA769070 and BioSample accession number SAMN22084215. Paired-end reads belonging to the fosmid pCC1FOS have been removed and the resulting six and a half million reads have been assembled.

## Supplementary Materials

The following are available online at https://www.mdpi.com/article/10.3390/microorganisms9112201/s1, Table S1: Identification of antibiotic resistance genes (ARGs) in three gallbladder metagenome samples (H-04, H-05 and H-06) and a fosmid library mapped against Resfinder and Comprehensive Antibiotic Resistance Database (CARD). Accession codes, as well as specific antibiotic resistance for each ARG, are provided. ARGs abundance is calculated by dividing the number of ARGs conferring resistance to certain groups of antibiotics by the number of sequence megabases. Figure S1: Structural conformation of novel β-galactosidase 2 found in the metagenomic dark matter fraction of gut microbiota. Figure S2: Redocking results of novel β-galactosidase 2, annotated in the metagenomic dark matter fraction of gut metagenomes, to several substrates: (A) ortho-nitrophenyl glucoside (ONPG), (B) lactose and C) 4’galactosyl-lactose. The similarity between the docked and bound conformations of substrates in the enzyme structure is illustrated. Colour codes are assigned to different atoms present in substrates: green (carbon), hydrogen (white), oxygen (red) and nitrogen (blue). It should be noted that this Figure illustrates the superimposition of two structures (docked and redocked). Superimposed docked and redocked conformation are very similar, highlighting the reproducibility of molecular docking simulations.

## References

[1] Molinero N., Ruiz L., Milani C., Gutiérrez-Díaz I., Sánchez B., Mangifesta M., Segura J., Cambero I., Campelo A.B., García-Bernardo C.M. (2019). The human gallbladder microbiome is related to the physiological state and the biliary metabolic profile. Microbiome.

[2] Song X., Wang X.A., Hu Y., Li H., Ren T., Li Y., Liu L., Li L., Li X., Wang Z. (2020). A metagenomic study of biliary microbiome change along the cholecystitis-carcinoma sequence. Clin. Transl. Med..

[3] Shen H., Ye F., Xie L., Yang J., Li Z., Xu P., Meng F., Li L., Chen Y., Bo X. (2015). Metagenomic sequencing of bile from gallstone patients to identify different microbial community patterns and novel biliary bacteria. Sci. Rep..

[4] Bernard G., Pathmanathan J.S., Lannes R., Lopez P., Bapteste E. (2018). Microbial dark matter investigations: How microbial studies transform biological knowledge and empirically sketch a logic of scientific discovery. Genome Biol. Evol..

[5] Bowman J.S. (2018). Identification of microbial dark matter in antarctic environments. Front. Microbiol..

[6] Oh J., Byrd A.L., Deming C., Conlan S., Kong H.H., Segre J.A. (2014). Biogeography and individuality shape function in the human skin metagenome. Nature.

[7] Tirosh O., Conlan S., Deming C., Lee-Lin S.Q., Huang X., Su H.C., Freeman A.F., Segre J.A., Kong H.H. (2018). Expanded skin virome in DOCK8-deficient patients. Nat. Med..

[8] Zamkovaya T., Foster J.S., de Crécy-Lagard V., Conesa A. (2021). A network approach to elucidate and prioritize microbial dark matter in microbial communities. ISME J..

[9] Lobb B., Kurtz D.A., Moreno-Hagelsieb G., Doxey A.C. (2015). Remote homology and the functions of metagenomic dark matter. Front. Genet..

[10] Sánchez-Flores A., Pérez-Rueda E., Segovia L. (2007). Protein homology detection and fold inference through multiple alignment entropy profiles. Proteins.

[11] Michalska K., Steen A.D., Chhor G., Endres M., Webber A.T., Bird J., Loyd K.G., Joachimiak A. (2015). New aminopeptidase from “microbial dark matter” archaeon. FASEB J..

[12] Sabater C., Blanco-Doval A., Margolles A., Corzo N., Montilla A. (2021). Artichoke pectic oligosaccharide characterisation and virtual screening of prebiotic properties using in silico colonic fermentation. Carbohydr. Polym..

[13] Quijada N.M., Rodríguez-Lázaro D., Eiros J.M., Hernández M. (2019). TORMES: An automated pipeline for whole bacterial genome analysis. Bioinformatics.

[14] Bortolaia V., Kaas R.S., Ruppe E., Roberts M.C., Schwarz S., Cattoir V., Philippon A., Allesoe R.L., Rebelo A.R., Florensa A.F. (2020). ResFinder 4.0 for predictions of phenotypes from genotypes. J. Antimicrob. Chemother..

[15] Alcock B.P., Raphenya A.R., Lau T.T., Tsang K.K., Bouchard M., Edalatmand A., Huynh W., Nguyen A.L.V., Cheng A.A., Liu S. (2020). CARD 2020: Antibiotic resistome surveillance with the comprehensive antibiotic resistance database. Nucleic Acids Res..

[16] Rho M., Tang H., Ye Y. (2010). FragGeneScan: Predicting genes in short and error-prone reads. Nucleic Acids Res..

[17] Mistry J., Chuguransky S., Williams L., Qureshi M., Salazar G.A., Sonnhammer E.L., Tosatto S.C.E., Paladin L., Rai S., Richardson L.J. (2021). Pfam: The protein families database in 2021. Nucleic Acids Res..

[18] Mistry J., Finn R.D., Eddy S.R., Bateman A., Punta M. (2013). Challenges in homology search: HMMER3 and convergent evolution of coiled-coil regions. Nucleic Acids Res..

[19] Saier Jr M.H., Reddy V.S., Moreno-Hagelsieb G., Hendargo K.J., Zhang Y., Iddamsetty V., Lam K.J.K., Tian N., Russum S., Wang J. (2021). The Transporter Classification Database (TCDB): 2021 update. Nucleic Acids Res..

[20] Kovatcheva-Datchary P., Nilsson A., Akrami R., Lee Y.S., De Vadder F., Arora T., Hallen A., Martens E., Björck I., Bäckhed F. (2015). Dietary fiber-induced improvement in glucose metabolism is associated with increased abundance of Prevotella. Cell Metab..

[21] Sabater C., Ruiz L., Margolles A. (2021). A Machine Learning Approach to Study Glycosidase Activities from Bifidobacterium. Microorganisms.

[22] Uritskiy G.V., DiRuggiero J., Taylor J. (2018). MetaWRAP—A flexible pipeline for genome-resolved metagenomic data analysis. Microbiome.

[23] Lu S., Wang J., Chitsaz F., Derbyshire M.K., Geer R.C., Gonzales N.R., Gwadz M., Hurwitz D.I., Marchler G.H., Song J.S. (2020). CDD/SPARCLE: The conserved domain database in 2020. Nucleic Acids Res..

[24] Fu L., Niu B., Zhu Z., Wu S., Li W. (2012). CD-HIT: Accelerated for clustering the next-generation sequencing data. Bioinformatics.

[25] Edgar R.C. (2004). MUSCLE: Multiple sequence alignment with high accuracy and high throughput. Nucleic Acids Res..

[26] Steinegger M., Meier M., Mirdita M., Vöhringer H., Haunsberger S.J., Söding J. (2019). HH-suite3 for fast remote homology detection and deep protein annotation. BMC Bioinform..

[27] Waterhouse A., Bertoni M., Bienert S., Studer G., Tauriello G., Gumienny R., Heer F.T., de Beer T.A.P., Rempfer C., Bordoli L. (2018). SWISS-MODEL: Homology modelling of protein structures and complexes. Nucleic Acids Res..

[28] Tian W., Chen C., Lei X., Zhao J., Liang J. (2018). CASTp 3.0: Computed atlas of surface topography of proteins. Nucleic Acids Res..

[29] Trott O., Olson A.J. (2010). AutoDock Vina. Improving the speed and accuracy of docking with a new scoring function, efficient optimization, and multithreading. J. Comput. Chem..

[30] Kumari R., Kumar R., Lynn A., Open Source Drug Discovery Consortium (2014). g_mmpbsa-A GROMACS tool for high-throughput MM-PBSA calculations. J. Chem. Inf. Model..

[31] Abraham M.J., Murtola T., Schulz R., Páll S., Smith J.C., Hess B., Lindahl E. (2015). GROMACS: High performance molecular simulations through multi-level parallelism from laptops to supercomputers. SoftwareX.

[32] He X., Ahn J. (2014). Assessment of conjugal transfer of antibiotic resistance genes in Salmonella Typhimurium exposed to bile salts. J. Microbiol..

[33] Gipson K.S., Nickerson K.P., Drenkard E., Llanos-Chea A., Dogiparthi S.K., Lanter B.B., Hibbler R.M., Yonker L.M., Hurley B.P., Faherty C.S. (2020). The great ESKAPE: Exploring the crossroads of bile and antibiotic resistance in bacterial pathogens. Infect. Immun..

[34] Grattagliano I., Ciampi S.A., Portincasa P., Gracia-Sancho J., Salvadó J. (2017). Gallbladder disease: Relevance of oxidative stress. Gastrointestinal Tissue.

[35] Singh S., Verma N., Taneja N. (2019). The human gut resistome: Current concepts & future prospects. Indian J. Med. Res..

[36] Elkins C.A., Nikaido H. (2002). Substrate specificity of the RND-type multidrug efflux pumps AcrB and AcrD of Escherichia coli is determined predominately by two large periplasmic loops. J. Bacteriol..

[37] Lu J., Wang Y., Li J., Mao L., Nguyen S.H., Duarte T., Coin L., Bond P., Yuan Z., Guo J. (2018). Triclosan at environmentally relevant concentrations promotes horizontal transfer of multidrug resistance genes within and across bacterial genera. Environ. Int..

[38] Sistrunk J.R., Nickerson K.P., Chanin R.B., Rasko D.A., Faherty C.S. (2016). Survival of the fittest: How bacterial pathogens utilize bile to enhance infection. Clin. Microbiol. Rev..

[39] Huang X., Kocabas E., Hernick M. (2011). The activity and cofactor preferences of N-acetyl-1-D-myo-inosityl-2-amino-2-deoxy-α-D-glucopyranoside deacetylase (MshB) change depending on environmental conditions. J. Biol. Chem..

[40] Newton G.L., Buchmeier N., Fahey R.C. (2008). Biosynthesis and functions of mycothiol, the unique protective thiol of Actinobacteria. Microbiol. Mol. Biol. Rev..

[41] Yin Y.J., Wang B.J., Jiang C.Y., Luo Y.M., Jin J.H., Liu S.J. (2010). Identification and quantification of mycothiol in Actinobacteria by a novel enzymatic method. Appl. Microbiol. Biotechnol..

[42] Jothivasan V.K., Hamilton C.J. (2008). Mycothiol: Synthesis, biosynthesis and biological functions of the major low molecular weight thiol in actinomycetes. Nat. Prod. Rep..

[43] Robinson S.L., Piel J., Sunagawa S. (2021). A roadmap for metagenomic enzyme discovery. Nat. Prod. Rep..

[44] Huang X., Hernick M. (2014). Automated docking studies provide insights into molecular determinants of ligand recognition by N-acetyl-1-d-myo-inosityl-2-amino-2-deoxy-α-d-glucopyranoside deacetylase (MshB). Biopolymers.

[45] Lamprecht D.A., Muneri N.O., Eastwood H., Naidoo K.J., Strauss E., Jardine A. (2012). An enzyme-initiated Smiles rearrangement enables the development of an assay of MshB, the GlcNAc-Ins deacetylase of mycothiol biosynthesis. Org. Biomol. Chem..

[46] Metaferia B.B., Fetterolf B.J., Shazad-ul-Hussan S., Moravec M., Smith J.A., Ray S., Gutierrez-Lugo M.T., Bewley C.A. (2007). Synthesis of natural product-inspired inhibitors of Mycobacterium tuberculosis mycothiol-associated enzymes: The first inhibitors of GlcNAc-Ins deacetylase. J. Med. Chem..

[47] Rogers I.L., Gammon D.W., Naidoo K.J. (2013). Conformational preferences of plumbagin with phenyl-1-thioglucoside conjugates in solution and bound to MshB determined by aromatic association. Carbohydr. Res..

[48] Lordan C., Thapa D., Ross R.P., Cotter P.D. (2020). Potential for enriching next-generation health-promoting gut bacteria through prebiotics and other dietary components. Gut Microbes.

[49] Gao X., Wu J., Wu D. (2019). Rational design of the beta-galactosidase from Aspergillus oryzae to improve galactooligosaccharide production. Food Chem..

[50] Zhu J., Sun J., Tang Y., Xie J., Wei D. (2020). Expression, characterization and structural profile of a heterodimeric β-galactosidase from the novel strain Lactobacillus curieae M2011381. Process Biochem..

