# Supplementary material for: Functional Characterisation of Bile Metagenome: Study of Metagenomic Dark Matter
Source: Microorganisms. 2021 Oct 21;9(11):2201. doi: 10.3390/microorganisms9112201 (PMC8621414; doi:10.3390/microorganisms9112201)
Supplement: Supplementary file 1 [file microorganisms-09-02201-s001.zip › microorganisms-1356941-supplementary.pdf]

Supplementary Table S1. Identification of antibiotic resistance genes (ARGs) in three gallbladder metagenome samples (H-04, H-05 and H-06) and a fosmid library mapped against Resfinder and Comprehensive Antibiotic Resistance Database (CARD). Accession codes as well as specific antibiotic resistance for each ARG are provided. ARGs abundance is calculated by dividing the number of ARGs conferring resistance to certain groups of antibiotics by the number of sequence megabases.

Sample	Database	Gene	Number of ARGs/Mb	Resistance
H-04	CARD	Erm(49)	0.11	Lincosamide, macrolide, streptogramin
H-04	CARD	TEM-116	0.11	Cephalosporin, monobactam, penam, penem
H-04	CARD; Resfinder	tet(Q); tet(W)	0.45	Doxycycline, tetracycline, minocycline
H-04	Resfinder	blaTEM-116	0.11	Amoxicillin, ampicillin, cephalothin, piperacillin, ticarcillin
H-04	Resfinder	cat(pC194)	0.11	Chloramphenicol
H-05	CARD	APH(3')-IIa	0.26	Aminoglycoside
H-05	CARD; Resfinder	Tet(O); tet(W); tet(W)	1.54	Doxycycline, tetracycline, minocycline
H-06	Resfinder	cat(pC194)	3.18	Chloramphenicol
Fosmid	CARD	acrB; acrE; acrF; acrS; H-NS	0.38	Cephalosporin, cephamycin, fluoroquinolone, glycylcycline, macrolide, penam, phenicol, rifamycin, tetracycline, triclosan
Fosmid	CARD	acrD	0.08	Aminoglycoside
Fosmid	CARD	baeR; baeS; cpxA; mdtA; mdtB; mdtC; tolC	0.54	Aminocoumarin, aminoglycoside, carbapenem, cephalosporin, cephamycin, fluoroquinolone, glycylcycline, macrolide, penam, penem, phenicol, rifamycin, tetracycline, triclosan
Fosmid	CARD	catI	0.08	Phenicol
Fosmid	CARD	CRP; emrA; emrB; emrR; evgA; evgS; gadW; gadX; mdtE; mdtF; mdtH	0.84	Fluoroquinolone, macrolide, penam, tetracycline
Fosmid	CARD	dfrA1	0.08	Diaminopyrimidine
Fosmid	CARD	emrK; emrY	0.15	Tetracycline
Fosmid	CARD	kdpE	0.08	Aminoglycoside
Fosmid	CARD	marA	0.08	Carbapenem, cephalosporin, cephamycin, fluoroquinolone, glycylcycline, monobactam, penam, penem, phenicol, rifamycin, tetracycline, triclosan
Fosmid	CARD	mdtG	0.08	Fosfomycin
Fosmid	CARD	mel	0.08	Lincosamide, macrolide, oxazolidinone, phenicol, pleuromutilin, streptogramin, tetracycline
Fosmid	CARD	mphB	0.08	Macrolide
Fosmid	CARD	msbA	0.08	Nitroimidazole
Fosmid	Resfinder	catA1	0.08	Chloramphenicol
Fosmid	Resfinder	dfrA1	0.08	Trimethoprim
Fosmid	Resfinder	mef(A)	0.08	Erythromycin, azithromycin

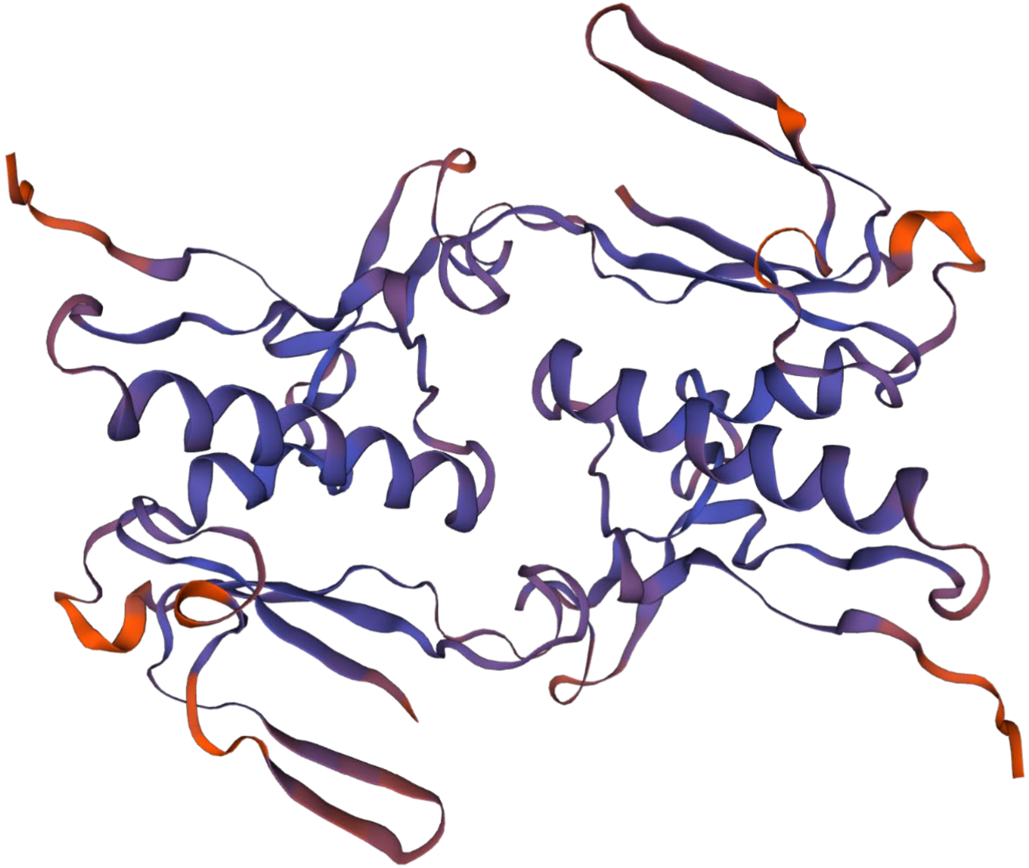

Supplementary Figure S1. Structural conformation of novel β-galactosidase 2 found in the metagenomic dark matter fraction of gut microbiota.

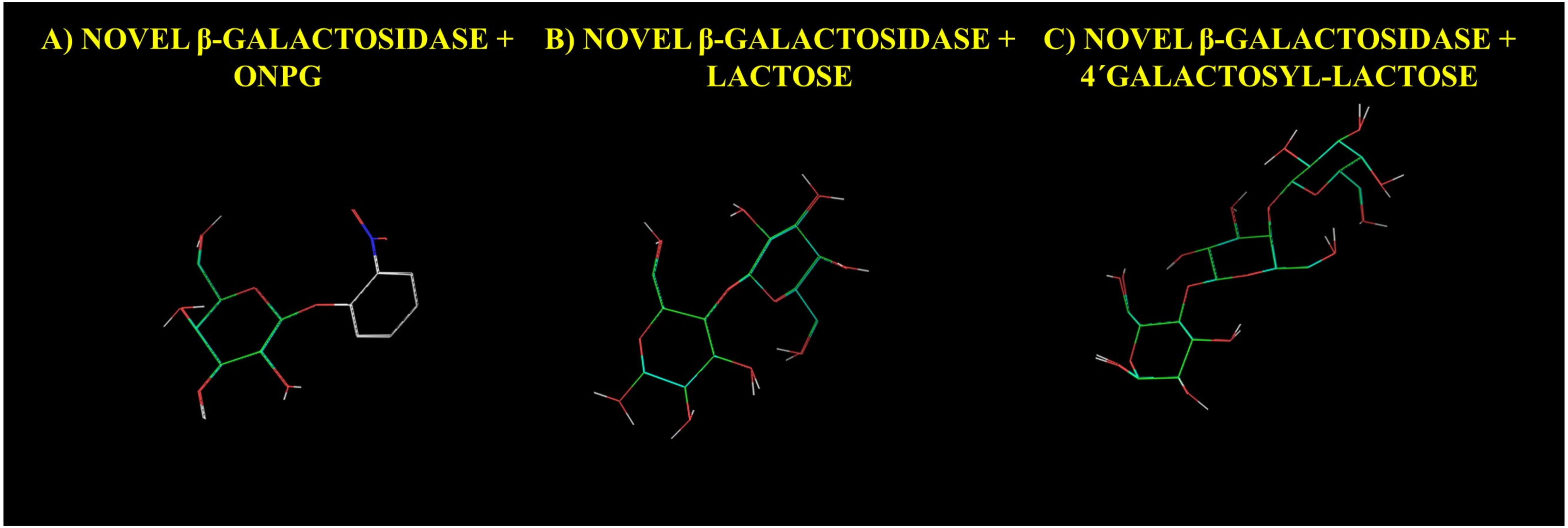

Supplementary Figure S2. Redocking results of novel β-galactosidase 2, annotated in the metagenomic dark matter fraction of gut metagenomes, to several substrates: A) ortho-nitrophenyl glucoside (ONPG), B) lactose, C) 4´galactosyl-lactose. The similarity between the docked and bound conformations of substrates in the enzyme structure is illustrated. Color codes are assigned to different atoms present in substrates: green (carbon), hydrogen (white), oxygen (red) and nitrogen (blue). It should be noted that this Figure illustrates the superimposition of two structures (docked and redocked). Superimposed docked and redocked conformation are very similar, highlighting the reproducibility of molecular docking simulations.
